# Direct oral anticoagulants in very elderly and high-bleeding-risk patients with atrial fibrillation often excluded from oral anticoagulation therapy: a nationwide population-based cohort study

**DOI:** 10.1093/europace/euaf230

**Published:** 2025-09-29

**Authors:** Young-Hae Go, So-Ryoung Lee, Eue-Keun Choi, Hyo-Jeong Ahn, Kyung-Do Han, Seil Oh, Gregory Y H Lip

**Affiliations:** Department of Internal Medicine, Seoul National University College of Medicine, 103 Daehak-ro, Jongno-gu, Seoul 03080, Republic of Korea; Department of Internal Medicine, Seoul National University College of Medicine, 103 Daehak-ro, Jongno-gu, Seoul 03080, Republic of Korea; Department of Internal Medicine, Seoul National University Hospital, 101 Daehak-ro, Jongno-gu, Seoul 03080, Republic of Korea; Department of Internal Medicine, Seoul National University College of Medicine, 103 Daehak-ro, Jongno-gu, Seoul 03080, Republic of Korea; Department of Internal Medicine, Seoul National University Hospital, 101 Daehak-ro, Jongno-gu, Seoul 03080, Republic of Korea; Department of Internal Medicine, Seoul National University Hospital, 101 Daehak-ro, Jongno-gu, Seoul 03080, Republic of Korea; Statistics and Actuarial Science, Soongsil University, Seoul, Republic of Korea; Department of Internal Medicine, Seoul National University College of Medicine, 103 Daehak-ro, Jongno-gu, Seoul 03080, Republic of Korea; Department of Internal Medicine, Seoul National University Hospital, 101 Daehak-ro, Jongno-gu, Seoul 03080, Republic of Korea; Department of Internal Medicine, Seoul National University College of Medicine, 103 Daehak-ro, Jongno-gu, Seoul 03080, Republic of Korea; Liverpool Centre for Cardiovascular Science, University of Liverpool and Liverpool Chest and Heart Hospital, Liverpool, UK; Department of Clinical Medicine, Aalborg University, Aalborg, Denmark

**Keywords:** Atrial fibrillation, Very elderly, Direct oral anticoagulant, Stroke, Bleeding

## Abstract

**Aims:**

In the Edoxaban Low-Dose for Elder Care Atrial Fibrillation Patients (ELDERCARE-AF) trial, very low-dose edoxaban (15 mg once daily) showed better efficacy and positive net clinical benefit compared with placebo in very elderly, high-bleeding-risk patients with atrial fibrillation (AF). However, there are limited data to generalize these results into daily practice. We aimed to investigate the optimal oral anticoagulant (OAC) strategy for the best net clinical benefit in ELDERCARE-AF-like patients.

**Methods and results:**

We used the Korean nationwide claims database to identify AF patients aged ≥80 years from 2014 to 2017 who had one or more ELDERCARE-AF trial inclusion criteria. The risks of ischaemic stroke, major bleeding, all-cause death, and net clinical outcome were evaluated. Primarily, we compared patients without OAC therapy (non-OAC group) to patients with direct OAC (DOAC) therapy (DOAC group), using a propensity score weighting method. A total of 23 858 patients were included (no OAC: 16 575; warfarin: 2390; DOAC: 4893). Among the DOAC group, 69% used low dose, and 9% used very low dose. Compared with the non-OAC group, the DOAC group showed lower risks of ischaemic stroke (hazard ratio, 95% confidence interval: 0.81, 0.68–0.96) and all-cause death (0.91, 0.86–0.96) and a higher risk of major bleeding (1.44, 1.21–1.70). The DOAC group was associated with a lower risk of net clinical outcome compared with the non-OAC group (0.93, 0.88–0.98).

**Conclusion:**

In very elderly, high-bleeding-risk patients with AF, DOACs that were prescribed in usual clinical practice showed better effectiveness and positive net clinical benefit compared with no OAC treatment.

## Graphical Abstract

**Graphical Abstract:**
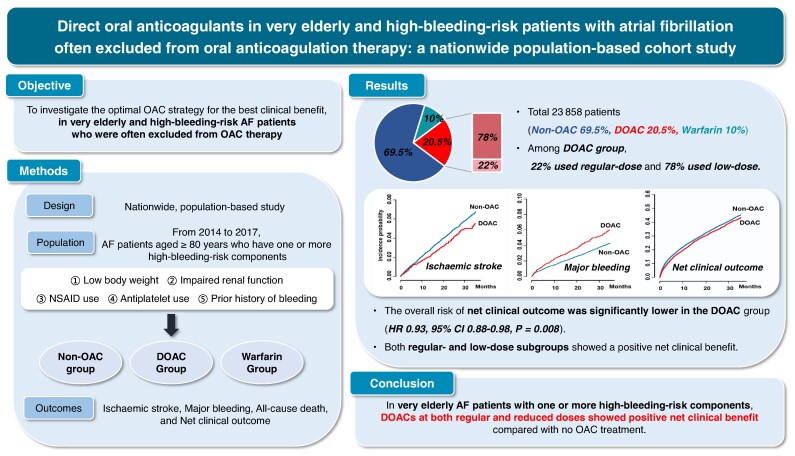
AF, atrial fibrillation; CI, confidence interval; DOAC, direct oral anticoagulant; HR, hazard ratio; NSAID, non-steroidal anti-inflammatory drug; OAC, oral anticoagulant.

What’s new?

- In very elderly atrial fibrillation (AF) patients with high bleeding risk, direct oral anticoagulants (DOACs) showed lower risks of stroke and all-cause death, compared with no oral anticoagulant (OAC).
- Direct oral anticoagulant was associated with a higher risk of major bleeding, compared with no OAC.
- Both regular-dose and low-dose DOAC regimens showed a positive net clinical benefit compared with no OAC.
- More proactive DOAC use can be considered in very elderly AF patients with high bleeding risk, often excluded from OAC therapy.

## Introduction

Atrial fibrillation (AF) is a frequently occurring cardiac arrhythmia with a rising prevalence and incidence, largely driven by an ageing population.^1^ Over time, the average age of patients with AF has consistently increased, and the proportion of elderly patients has continuously increased among AF patients.^1,2^ These elderly AF patients often exhibit frailty due to factors such as multiple comorbidities, polypharmacy, and low body weight.^3^ Their heightened risk of bleeding has made physicians hesitant to prescribe oral anticoagulants (OACs) for stroke prevention.^4,5^

Of note, very elderly patients (i.e. those aged 80 years and older) at high risk of bleeding have often been underrepresented in pivotal randomized controlled trials (RCTs) of direct OACs (DOACs).^6^ In a landmark trial comparing edoxaban with warfarin, 17% of participants were aged 80 or older.^7,8^

The Low-Dose Edoxaban in Very Elderly Patients with Atrial Fibrillation (ELDERCARE-AF) trial specifically focused on very elderly AF patients at high risk of bleeding who were not suitable for usual-dose DOAC therapy.^9^ This trial demonstrated that a very low-dose regimen of edoxaban (once-daily edoxaban 15 mg) significantly reduced stroke risk with no significant increase in major bleeding compared with placebo.

However, still, there are some challenges in generalizing the results of the ELDERCARE-AF trial into daily clinical practice. This trial exclusively evaluated a specific very low-dose regimen of edoxaban, limiting its relevance for assessing the efficacy and safety of usual-dose DOAC regimens.^10^ Recent observational studies suggest that on-label reduced dose of DOAC may provide comparable efficacy to very low-dose regimens in very elderly AF patients.^11,12^ Nevertheless, comparisons between usual-dose DOAC regimens and non-OAC treatment in this population remain limited.

In this study, we aimed to investigate the optimal OAC strategy to achieve the best net clinical benefit in very elderly AF patients at high risk of bleeding who were not suitable for usual-dose DOACs (i.e. ELDERCARE-AF-like patients).

## Methods

### Data source and study design

This retrospective nationwide observational study utilized the Korean National Health Insurance Service (NHIS) database, a mandatory government-provided health insurance programme. The database contains comprehensive claims data, including demographics, diagnoses, health examination data, prescription records, and medical expenses.^13,14^ Diagnoses were ascertained through the *International Classification of Diseases*, 10th Revision, Clinical Modification (ICD-10-CM). The NHIS approved the use of its anonymized database following a review of the submitted study proposal. As the data were fully anonymized and retrospective, individual patient consent was not required. The Institutional Review Board of Seoul National University Hospital approved this study (No. E-2502-009-1609).

**Figure 1:**
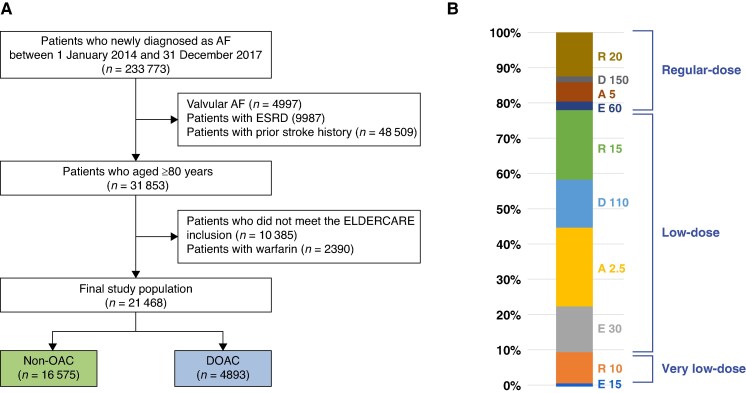
Study enrolment flow and detailed dose distribution in the DOAC group. (*A*) Study enrolment flow. (*B*) Detailed dose distribution in the DOAC group. A, apixaban; AF, atrial fibrillation; D, dabigatran; DOAC, direct oral anticoagulant; E, edoxaban; ESRD, end-stage renal disease; OAC, oral anticoagulant; R, rivaroxaban.

The enrolment flow is illustrated in *Figure 1A*. Between January 2014 and December 2017, patients aged 80 years or older with newly diagnosed non-valvular AF were identified. Exclusion criteria included end-stage renal disease, for which DOACs were generally not recommended, and a prior history of stroke. Among these patients, those with one or more of the following modified ELDERCARE-AF-like population enrolment criteria were finally included in the analysis: (i) low body weight (≤45 kg); (ii) impaired renal function defined as low estimated glomerular filtration rate (eGFR, 15–30 mL/min/m^2^) or clinical chronic kidney disease (CKD) diagnosis; (iii) concomitant use of non-steroidal anti-inflammatory drugs (NSAIDs); (iv) concomitant use of antiplatelet agents; and (v) prior history of bleeding including intracranial haemorrhage, gastrointestinal bleeding, or other critical organ bleeding (*Table 1*).

**Table 1:** ELDERCARE-AF inclusion criteria of study population

	Present study	ELDERCARE-AF study
Number	21 468	984
Age ≥80 years	100%	100%
1 or more additional factors among as follows		
(1) Body weight ≤45 kg	21.7%^a^	38.0%
(2) Renal impairment (eGFR 15–30 mL/min/1.73 m^2^ or CKD diagnosis)	18.1%	41.0%
(3) Concomitant use of NSAID	25.0%	32.2%
(4) Concomitant use of antiplatelet agent	56.1%	53.8%
(5) Prior history of bleeding		
(5-1) Prior intracranial haemorrhage	1.2%	8.1%
(5-2) Prior gastrointestinal bleeding	5.9%	12.9%
(5-3) Prior other critical organ bleeding	0.8%	2.1%

CKD, chronic kidney disease; eGFR, estimated glomerular filtration rate; NSAID, non-steroidal anti-inflammatory drug.

^a^The proportion was calculated among patients with available health examination data.

A total of 23 858 patients meeting ELDERCARE-AF-like criteria were included in this study, comprising 16 575 patients prescribed DOACs, 2390 prescribed warfarin, and 4893 who did not receive any OAC (non-OAC group). For the main analysis, 21 468 patients were included after excluding those prescribed warfarin.

### Covariates

Patient demographic data and comorbidities such as hypertension, diabetes, heart failure, and CKD were obtained from the NHIS database. Stroke risk was assessed using the CHA_2_DS_2_-VASc score, and comorbidity burden was quantified with the Charlson comorbidity index, both calculated based on covariate information.^15,16^ Detailed definitions of comorbidities and scoring systems are provided in Supplementary material online, *Tables S1*–*S3*. For patients with available health examination data, additional variables such as body weight, eGFR, smoking status, alcohol consumption, and physical activity were analysed.

### Outcomes and follow-up

To evaluate the effectiveness and safety of treatment groups, the risks of ischaemic stroke, major bleeding, all-cause death, and the net clinical outcome defined as the composite clinical outcome of ischaemic stroke, major bleeding, and all-cause death were assessed during follow-up. Patients were censored at the first occurrence of any predefined outcomes, death, or the conclusion of the study period (31 December 2018), which ever came first. Detailed operational definitions of study outcomes are summarized in Supplementary material online, *Table S1*.

### Statistical analysis

For continuous variables, data were reported as mean ± standard deviation or median [interquartile range (IQR)], whereas categorical variables were represented as number and percentage. To minimize baseline differences between the two treatment groups, the inverse probability of treatment weighting (IPTW) method was applied.^17–19^ Propensity scores were calculated using logistic regression, based on baseline covariates such as age, sex, CHA_2_DS_2_-VASc score, frailty index,^20,21^ Charlson comorbidity index, and comorbidities including hypertension, diabetes, heart failure, dyslipidaemia, prior myocardial infarction, chronic obstructive pulmonary disease, peripheral artery disease, and cancer. After applying IPTW, balance between groups was evaluated using absolute standardized differences (ASDs), with an ASD ≤ 0.1 indicating acceptable balance.^22^ Weighted incidence rates of study outcomes were calculated as the weighted number of events per 100 person-years at risk. Kaplan–Meier survival analysis with log-rank tests and weighted Cox proportional hazards models were used to compare clinical outcomes, presenting hazard ratios (HRs) and 95% confidence intervals (CIs). Statistical significance was considered for a *P*-value of <0.05. All analyses were performed with SAS 9.4.

### Subgroup and exploratory analyses

Criteria specific to ELDERCARE-AF-like population, such as body weight, renal impairment, use of concomitant NSAID or antiplatelet agents, and previous bleeding history, were not included in the propensity score calculation. Instead, we performed subgroup analyses not only based on age, sex, and CHA_2_DS_2_-VASc score but also on factors included in the ELDERCARE-like-AF population criteria. Multivariable Cox proportional hazards models were employed, adjusting covariates included in the propensity score calculation in the main IPTW analysis. Statistical significance for subgroup differences was assessed using a *P*-for-interaction (<0.05).

As part of exploratory analyses, differences in ischaemic stroke, major bleeding, and net clinical outcome were evaluated based on the dose of DOAC, categorized into regular and low doses. Regular dose included dabigatran 150 mg twice daily, rivaroxaban 20 mg once daily, edoxaban 60 mg once daily, and apixaban 5 mg twice daily. Low dose included dabigatran 110 mg twice daily, rivaroxaban 15 and 10 mg once daily, edoxaban 30 and 15 mg once daily, and apixaban 2.5 mg twice daily. Additionally, all study outcomes were also analysed according to the type of DOAC used.

To evaluate the effectiveness and safety of warfarin compared with the non-OAC group, an additional exploratory analysis was conducted by comparing warfarin users, who had been excluded from the main analysis. Baseline differences were addressed using the IPTW method, and balance between groups was assessed with ASD.^17,18,22^ After IPTW, the risks of ischaemic stroke, major bleeding, death, and net clinical outcome were analysed using weighted Cox proportional hazards models.

## Results

### Baseline characteristics

A total of 23 858 patients were included in this study (non-OAC, *n* = 16 575; warfarin, *n* = 2390; and DOACs, *n* = 4893). Among the DOAC group, 69% used a low-dose regimen, including rivaroxaban 15 mg once daily, dabigatran 110 mg twice daily, apixaban 2.5 mg twice daily, and edoxaban 30 mg once daily, while 9% used a very low-dose regimen, including rivaroxaban 10 mg once daily and edoxaban 15 mg once daily (*Figure 1B*).

After excluding warfarin users, 21 468 patients were analysed in the main analysis, categorized into non-OAC and DOAC groups. The composition of ELDERCARE-AF-like inclusion criteria in the study population is shown in *Table 1*. Among patients with available data, 21.7% met the criterion for low body weight, and 18.1% met the criterion for renal impairment. Both proportions were lower than those reported in the ELDERCARE-AF trial. The proportion of patients using antiplatelet agents concurrently was 56.1%, similar to 53.8% in ELDERCARE-AF. Baseline characteristics of the total study population are summarized in Supplementary material online, *Table S4*.

The baseline characteristics of the non-OAC group and DOAC group before and after IPTW are presented in *Table 2*. Before IPTW, compared with the DOAC group, the non-OAC group was slightly older (85.4 ± 4.4 vs. 84.9 ± 4.0 years), had lower CHA_2_DS_2_-VASc score (4.4 ± 1.1 vs. 4.6 ± 1.1), and showed fewer comorbidities such as hypertension (80.3 vs. 84.6%), heart failure (37.7 vs. 51.6%), and dyslipidaemia (36.9 vs. 43.1%). After applying IPTW, all covariates were well balanced between the groups (ASD ≤ 0.1). The weighted population had a mean age of 85.3 years and a mean CHA_2_DS_2_-VASc score of 4.4.

**Table 2:** Baseline characteristics of non-OAC group and DOAC group before and after IPTW

	Pre-IPTW			Post-IPTW		
	Non-OAC	DOAC	ASD	Non-OAC	DOAC	ASD
Number	16 575	4893		16 576.2	4893	
Age, years (mean ± SD)	85.4 ± 4.4	84.9 ± 4.0	0.117	85.3 ± 4.4	85.3 ± 4.2	0.003
Age, years (median, IQR)	84 (82–88)	85 (82–88)		84 (82–88)	84 (82–88)	
Age						
80 < age ≤ 85	50.0	53.7		51.0	50.1	
85 < age ≤ 90	32.1	32.2		31.8	33.3	
≥90	17.9	14.1		17.2	16.6	
Female	63.4	66.0	0.053	64.0	64.2	0.003
Charlson comorbidity score (mean ± SD)	3.9 ± 2.4	4.0 ± 2.3	0.049	3.9 ± 2.4	3.9 ± 2.3	0.004
>3	51.0	53.0		51.5	51.6	
Frailty index (mean ± SD)	9.4 ± 7.2	8.9 ± 6.9	0.071	9.3 ± 7.2	9.3 ± 7.1	0.038
CHA_2_DS_2_-VASc score (mean ± SD)	4.4 ± 1.1	4.6 ± 1.1	0.190	4.4 ± 1.1	4.4 ± 1.1	0.011
CHA_2_DS_2_-VASc score (median, IQR)	4 (4–5)	5 (4–5)		4 (4–5)	4 (4–5)	
CHA_2_DS_2_-VASc score						
2	4.9	2.6		4.5	3.6	
3	17.3	13.6		16.4	16.6	
4	31.7	29.9		31.2	32.0	
5	30.3	33.7		31.2	30.9	
6	13.3	17.1		14.1	14.4	
≥7	2.5	3.1		2.7	2.6	
Comorbidities						
Hypertension	80.3	84.6	0.113	81.3	81.9	0.014
Diabetes mellitus	23.3	23.8	0.013	23.4	23.5	0.003
Heart failure	37.7	51.6	0.281	40.9	41.1	0.003
Dyslipidaemia	36.9	43.1	0.125	38.4	38.7	0.006
Prior myocardial infarction	8.1	7.3	0.029	8.0	8.1	0.005
Chronic kidney disease	18.6	16.13	0.066	18.8	15.7	0.082
Peripheral artery disease	26.6	27.5	0.018	26.8	27.0	0.004
Chronic obstructive pulmonary disease	12.8	13.8	0.029	13.0	13.0	<0.001
Cancer	8.1	7.9	0.004	8.0	8.0	0.001
Low income	16.0	15.1	0.026	16.0	15.3	0.019
Patients with health examination data^a^	45.4	41.2				
eGFR (mean ± SD, mL/min/1.73 m^2^)	72.7 ± 39.9	73.8 ± 28.4	0.033	72.5 ± 39.8	74.3 ± 29.9	0.050
Body weight (mean ± SD, kg)	55.4 ± 11.1	56.2 ± 11.5	0.069	55.4 ± 11.1	56.1 ± 11.4	0.057
≤45 kg	21.6	21.9		21.4	22.4	
45 < body weight ≤ 60 kg	46.5	42.9		46.6	42.7	
>60 kg	31.9	35.2		32.0	35.0	
Smoking			0.023			0.002
Non-smoker	76.6	77.6		76.9	76.8	
Ex-smoker	17.0	16.5		16.8	17.1	
Current smoker	6.4	5.9		6.3	6.0	
Alcohol			0.006			0.006
Non	84.9	85.2		85.1	84.8	
Mild to moderate	13.1	12.8		13.0	13.2	
Heavy drinker	2.0	2.0		2.0	2.0	
Performing regular exercise	11.4	11.4	<0.001	11.3	11.5	0.006

Continuous variables are shown as mean and standard deviation. Categorical variables are presented as percentages.

ASD, absolute standardized difference; DOAC, direct oral anticoagulant; eGFR, estimated glomerular filtration rate; IPTW, inverse probability of treatment weighting; IQR, interquartile range; OAC, oral anticoagulant; SD, standard deviation.

^a^These variables were calculated among patients with available health examination data.

### Primary analysis: comparison between direct oral anticoagulant and non-oral anticoagulant groups

**Figure 2:**
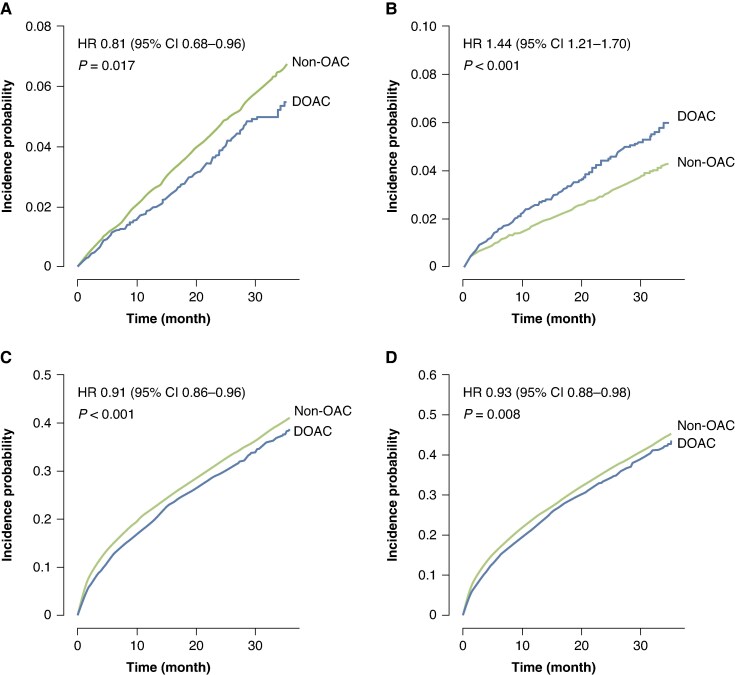
Weighted cumulative incidence curves of ischaemic stroke, major bleeding, all-cause death, and net clinical outcome. (*A*) Ischaemic stroke. (*B*) Major bleeding. (*C*) All-cause death. (*D*) Net clinical outcome. CI, confidence interval; DOAC, direct oral anticoagulant; HR, hazard ratio; OAC, oral anticoagulant.

**Figure 3:**
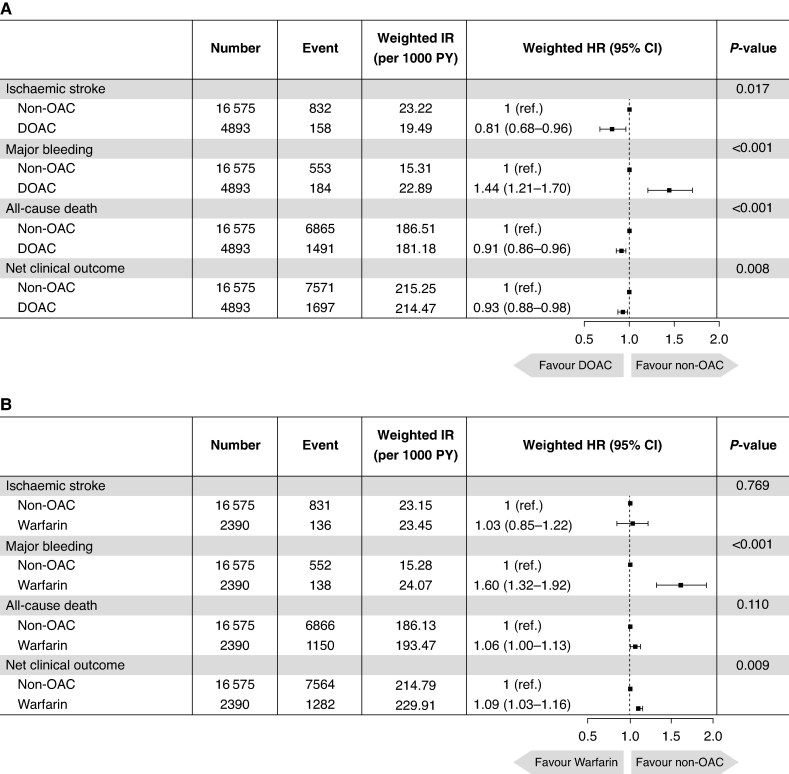
Clinical outcomes for DOAC group vs. non-OAC group and warfarin group vs. non-OAC group. (*A*) Clinical outcomes between DOAC group and non-OAC group. (*B*) Clinical outcomes between warfarin group and non-OAC group. CI, confidence interval; DOAC, direct oral anticoagulant; HR, hazard ratio; IR, incidence rate; OAC, oral anticoagulant; PY, person-years.

The median follow-up duration was 1.94 years (IQR, 1.08–3.08 years). Compared with the non-OAC group, the DOAC group was associated with a lower risk of ischaemic stroke (HR 0.81, 95% CI 0.68–0.96, *P* = 0.017) and all-cause death (HR 0.91, 95% CI 0.86–0.96, *P* < 0.001), but an increased risk of major bleeding (HR 1.44, 95% CI 1.21–1.70, *P* < 0.001). Despite the higher bleeding risk, DOAC use resulted in a lower risk of the net clinical outcome compared with the non-OAC treatment (HR 0.93, 95% CI 0.88–0.98, *P* = 0.008). The weighted cumulative incidence curves demonstrated significantly lower event rates in the DOAC group for ischaemic stroke, all-cause death, and the net clinical outcome, while showing significantly higher rates of major bleeding (*Figure 2*). The weighted incidence rates and HRs for study outcomes are presented in *Figure 3A*.

### Subgroup analysis

In subgroup analyses based on the ELDERCARE-AF criteria, most subgroups showed no significant interaction for the net clinical outcome (see Supplementary material online, *Figure S1*). A significant interaction was observed with concomitant antiplatelet use, whereby in the subgroup receiving antiplatelet therapy, the increased risk of net clinical outcome associated with DOAC use was more pronounced (*P*-for-interaction < 0.001).

### Direct oral anticoagulant type and dose

The distribution of prescribed DOACs by types was as follows: 2015 (41.2%) rivaroxaban, 1374 (28.1%) apixaban, 756 (15.5%) edoxaban, and 748 (15.3%) dabigatran. When comparing each DOAC group to the non-OAC group, rivaroxaban and dabigatran were associated with higher risks of major bleeding (HR 1.59, 95% CI 1.27–2.00 for rivaroxaban; HR 1.47, 95% CI 1.03–2.10 for dabigatran; *P* < 0.001), while edoxaban showed a significantly lower risk of net clinical outcome (HR 0.75, 95% CI 0.64–0.87, *P* < 0.001) (see Supplementary material online, *Figure S2*).

By DOAC doses, 1073 (21.9%) patients received regular-dose DOAC, while 3820 (78.1%) patients were prescribed low-dose DOAC. Compared with the non-OAC group, the low-dose regimen was associated with a lower risk of net clinical outcome (HR 0.91, 95% CI 0.86–0.97). Similarly, regular-dose DOACs were associated with a lower risk of net clinical outcome than the non-OAC group (HR 0.90, 95% CI 0.81–1.00) (see Supplementary material online, *Figure S3*).

### Exploratory analysis: comparison between warfarin and non-oral anticoagulant groups

Pre- and post-IPTW baseline characteristics of the warfarin group and non-OAC group are summarized in Supplementary material online, *Table S5*. After IPTW, the baseline characteristics were well balanced between the groups. Compared with the non-OAC group, the warfarin group was not associated with a lower risk of ischaemic stroke (HR 1.03, 95% CI 0.85–1.22, *P* = 0.769) or all-cause death (HR 1.06, 95% CI 1.00–1.13, *P* = 0.11). The warfarin group was associated with higher risks of major bleeding (HR 1.60, 95% CI 1.32–1.92, *P* < 0.001) and net clinical outcome (HR 1.09, 95% CI 1.03–1.16, *P* = 0.009) (*Figure 3B*).

## Discussion

This was a real-world, nationwide, population-based study evaluating the optimal OAC strategy for the best net clinical outcome in very elderly AF patients with one or more frail components. This study’s main findings were as follows: (i) In real-world clinical practice, 69.5% of ELDERCARE-AF-like patients did not receive OACs, while 20.5% were prescribed DOACs and 10% were treated with warfarin. Among the DOAC group, 69% used low-dose DOAC, while 9% were on very lose-dose DOAC. (ii) The DOAC group was associated with significantly lower risks of ischaemic stroke and all-cause death by ∼20 and 10%, respectively, but a higher risk of major bleeding by 43% compared with those not receiving OACs. The overall risk of net clinical outcome was significantly lower in the DOAC group. (iii) Compared with the non-OAC group, the warfarin group showed no significant differences in the risks of ischaemic stroke and all-cause death, but the risks of major bleeding and net clinical outcome were significantly higher in the warfarin group.

These findings suggest that even in very elderly AF patients with high bleeding risk, usual-dose DOAC regimens provide a positive net clinical outcome compared with non-OAC use. This aligns with previous studies showing that DOAC offers better outcomes than non-OAC use, even in frail or elderly AF patients.^23,24^ An observational study of frail AF patients aged 50 and older also demonstrated that OAC users had a net clinical benefit compared with non-OAC users,^23^ though the benefit declined with increasing age, leaving questions about the optimal OAC strategy for very elderly, frail patients.^23^ Another study of frail patients aged 65 and older showed similar results, demonstrating that DOACs reduced the risks of ischaemic stroke, major bleeding, and cardiovascular death compared with warfarin.^20^ Our study builds on those findings by showing that usual-dose DOACs (i.e. beyond the 15 mg edoxaban dose in the ELDERCARE-AF trial) also provide significant benefits in very elderly frail patients.

Current Asian AF guidelines emphasize that frailty alone should not be a reason to withhold anticoagulation and recommend individualized stroke and bleeding risk assessments.^25,26^ However, implementing this recommendation in real-world practice remains challenging. Our nationwide, observational cohort differed from the ELDERCARE-AF trial in several respects: The proportions of low body weight and moderate renal impairment were not identical, and frailty was measured using a claims-based Hospital Frailty Risk Score rather than the assessment tool employed in ELDERCARE-AF. Direct comparisons are therefore limited. In addition, observational studies can capture a more heterogeneous and often higher-risk patient population than randomized trials, including individuals who might otherwise be excluded from RCTs. Nonetheless, the consistent clinical benefits of DOACs observed in our broader cohort suggest that the ELDERCARE-AF findings are likely generalizable across a wider spectrum of frail elderly AF patients.

One unique aspect of this study is its comparison between warfarin and non-OAC groups, which was not addressed in the ELDERCARE-AF trial. Previous studies have shown that Asians face a higher bleeding risk with warfarin, which may explain the elevated bleeding risk in our warfarin group.^27–29^ At the same time, Asians tend to maintain a lower international normalized ratio due to bleeding concerns, and their time in therapeutic range is often less stable than in Western populations.^30,31^ These factors likely explain why, unlike DOACs, warfarin did not significantly reduce stroke risk and ultimately resulted in worse composite outcomes.

Regarding antiplatelet use, the subgroup receiving both OAC and antiplatelet agents showed a negative net clinical benefit, favouring the non-OAC group. In the ELDERCARE-AF trial, the efficacy of 15 mg edoxaban was attenuated in the patients receiving concomitant antiplatelet therapy.^9^ Previous randomized trials have also shown unfavourable outcomes with OAC–antiplatelet combinations,^32–34^ and our study demonstrated similar findings in very elderly patients at high bleeding risk. Given these results, caution is needed when prescribing antiplatelets in AF patients to optimize stroke prevention while minimizing bleeding risk. Particularly in high-bleeding-risk patients, clinicians should avoid unnecessary antiplatelet use.

Over the past decades, OAC strategies for AF patients have improved substantially. The introduction of DOACs has enhanced safety and expanded access to OAC therapy beyond the limitations of vitamin K antagonists.^35^ Nevertheless, challenges such as non-persistence and inappropriate underdosing remain in real-world settings, often leading to an increased risk of stroke.^35^ These issues are particularly prevalent among frail or elderly patients.^36–38^ Although evidence supporting the clinical benefits of DOACs in these high-risk patients is growing,^39,40^ they are still frequently underrepresented in OAC strategies and often remain undertreated.^41^ In our cohort, nearly 60% of patients in the ELDERCARE-like cohort remained without OAC therapy even 1 year after AF diagnosis (see Supplementary material online, *Figure S4*), highlighting a gap between growing evidence and real-world prescription patterns. This study supports the broader use of DOACs in routine clinical practice, even among populations that have traditionally been underrepresented in OAC strategies.

However, determining the ideal DOAC dose in elderly, high-risk populations remains challenging. In this study, ∼78% of DOAC users received a low-dose DOAC. Given that older age and higher CHA_2_DS_2_-VASc scores are associated with inappropriate DOAC dosing,^42^ a substantial portion of these patients may have been underdosed inappropriately. Previous Korean nationwide registry studies have shown that off-label underdosing is often observed in high-risk patients.^43,44^ While some studies suggest that the clinical hazard of off-label underdosing may not always be evident,^44^ other reports have demonstrated that off-label underdosing is associated with higher rates of adverse clinical events even among Asian populations.^43,45–47^ In the present study, due to the limited availability of data on body weight and renal function for all patients, we could not accurately determine whether DOAC prescriptions were on-label. Nonetheless, since a favourable net clinical benefit was observed in both the low-dose and regular-dose subgroups, our findings support the more active consideration of appropriate, on-label dosing when prescribing DOACs in this population.

### Limitations

Despite its strengths, our study has several limitations. First, although we adjusted for potential confounders, unmeasured variables may still exist due to the observational nature of the study. In real-world practice, physicians may avoid prescribing OACs to patients with limited life expectancy or perceived to be at high risk. As a result, while IPTW was used to minimize baseline differences, the non-OAC group may have included more patients at inherently higher bleeding risk, which could have introduced bias by indication. Secondly, as the analysis followed an intention-to-treat approach, treatment changes during follow-up may have influenced outcomes. However, this reflects real-world scenarios where treatment modifications occur, making our findings applicable to clinical practice. Thirdly, body weight and eGFR were unavailable for some patients, preventing the assessment of DOAC effectiveness and safety based on dosing label adherence. In Korea, off-label underdosing is reported in 27–36% of cases, likely higher in this elderly, frail population.^43,48^ Fourth, due to the nature of the NHIS data, we were unable to assess the severity of stroke events, such as using the National Institutes of Health Stroke Scale (NIHSS). Given that our cohort consisted of a high-risk elderly population, some of the strokes may have been disabling.^49^ Thus, the potential benefit of OACs could have meaningful implications for patients’ quality of life and functional status, beyond the reduction in stroke incidence alone. Fifth, we lacked direct comparisons among DOACs. While subgroup analysis suggested a greater net clinical benefit with edoxaban compared with the non-OAC group, the absence of direct DOAC comparisons limits conclusions on the most favourable choice. Further prospective studies are needed to explore this. Lastly, this study included only East Asians. Given recognized ethnic differences in AF-related complications (stroke, bleeding)^50,51^ and the so-called East Asian paradox,^52,53^ caution is required when generalizing these findings to other populations.

## Conclusions

In very elderly patients with AF who had one or more high-bleeding-risk components, DOACs at both regular and reduced doses showed better effectiveness and positive net clinical benefit compared with no OAC treatment. In contrast, warfarin did not show benefit compared with no OAC treatment in these very elderly frail patients with AF.

## Supplementary Material

euaf230_Supplementary_Data

## Data Availability

All data analysed during this study are available at the Korean National Health Insurance Data Sharing Service.
