# Supplementary material for: Direct oral anticoagulants in very elderly and high-bleeding-risk patients with atrial fibrillation often excluded from oral anticoagulation therapy: a nationwide population-based cohort study
Source: Europace. 2025 Sep 29;27(10):euaf230. doi: 10.1093/europace/euaf230 (PMC12510312; doi:10.1093/europace/euaf230)
Supplement: euaf230_Supplementary_Data [file euaf230_supplementary_data.docx]

**Net clinical benefit of direct oral anticoagulants in very elderly and high bleeding risk patients with atrial fibrillation who are often excluded from oral anticoagulation therapy: a nationwide population-based cohort study**

**Supplementary Tables**

**Supplementary Table 1. Definitions of covariates and outcomes**

**Supplementary Table 2. Definition of Charlson comorbidity index**

**Supplementary Table 3. Calculation of the hospital Frailty risk score. The score is an aggregate of 109 ICD-10 diagnostic codes that were found to be associated with frailty risk**

**Supplementary Table 4. Baseline characteristics of total study population**

**Supplementary Table 5. Baseline characteristics of no-OAC group and Warfarin group before and after IPTW**

**Supplementary Figures**

**Supplementary Figure 1. Subgroup analyses according to the age, sex, CHA₂DS₂-VASc score, GFR, previous history of GI bleeding, body weight, NSAID use, and antiplatelet agent use**

**Supplementary Figure 2. Hazard ratios of ischemic stroke, major bleeding, all-cause death, and net clinical outcome in 4 direct oral anticoagulants versus Non-OAC group**

**Supplementary Figure 3. Hazard ratios of ischemic stroke, major bleeding, all-cause death, and net clinical outcome by DOAC dose**

**Supplementary Figure 4. Anticoagulant prescription trends over one year in the ELDERCARE-AF-like cohort**

**Supplementary Table 1. Definitions of covariates and outcomes**

| **Diagnosis** | **ICD-10-CM code and definition** | **Diagnostic definition** |  |
| --- | --- | --- | --- |
| **Inclusion/exclusion criteria** | | |  |
| **Atrial fibrillation** | I48.0-I48.4, I48.9 | Admission or outpatient department ≥1 |  |
| **Valvular atrial fibrillation** | I05.0, I05.2, I05.9, Z95.2-Z95.4 | Admission or outpatient department ≥1 |  |
| **Prior stroke** | I63, I64 | Admission or outpatient department ≥1 |  |
| **End stage renal disease** | N18.5, Z49 | Dialysis ≥2 |  |
|  |  | Dialysis: hemodialysis (O7011-O7020), or peritoneal dialysis (O7017, O7075) |  |
| **Comorbidities** | | |  |
| **Hypertension** | I10-I13, I15; and minimum 1 prescription of anti-hypertensive drug (thiazide, loop diuretics, aldosterone antagonist, alpha-/beta-blocker, calcium-channel blocker, angiotensin-converting enzyme inhibitor, angiotensin II receptor blocker). | Admission ≥1 or outpatient department ≥2 |  |
| **Diabetes mellitus** | E11-E14; and minimum 1 prescription of anti-diabetic drugs (sulfonylureas, metformin, meglitinides, thiazolidinediones, dipeptidyl peptidase-4 inhibitors, α-glucosidase inhibitors, and insulin). | Admission ≥1 or outpatient department ≥2 |  |
| **Heart failure** | I50 | Admission or outpatient department ≥1 |  |
| **Dyslipidemia** | E78 | Admission or outpatient department ≥1 |  |
| **Prior myocardial infarction** | I21, I22 | Admission or outpatient department ≥1 |  |
| **Chronic kidney disease** | N18, N19 | Admission or outpatient department ≥1 |  |
| **Peripheral artery disease** | I70, I73 | Admission or outpatient department ≥1 |  |
| **Chronic obstructive pulmonary disease** | J41-44 | Admission ≥1 |  |
| **Cancer** | C00-97 and RID code (V193) | Admission or outpatient department ≥1 |  |
| **Alcohol** | Mild to moderate drinker: alcohol consumption >0g to <30g per day  Heavy drinker: alcohol consumption ≥30g per day | Based on the results of health examination |  |
| **Regular exercise** | Performing a moderate physical activity more than 30 minutes at least 5 times per week or strenuous physical activity more than 20 minutes at least 3 times per week. | Based on the results of health examination |  |
| **Low income** | Income lowest 20% and medical aid |  |  |
| **Scores** | | |  |
| **CHA_2_DS_2_-VASc score** | Heart failure (1 point), hypertension (1 point), age ≥75 years (2 points), diabetes (1 point), previous stroke/systemic embolism/transient ischemic attack (2 points), vascular disease (prior myocardial infarction or peripheral artery disease, 1 point) and female sex (1 point) | |  |
| **Charlson Comorbidity Index** | Supplementary Table 2 | |  |
| **Frailty index** | Supplementary Table 3 | |  |
| **Study outcomes of the main analysis** | | |  |
| **Ischemic stroke** | I63, I63 | Primary diagnosis, admission ≥1 (≥3 days) and brain imaging (CT or MRI) ≥1 |  |
| **Major bleeding** | Major gastrointestinal bleeding + Intracranial hemorrhage + Extracranial/unclassified major bleeding | Each definition was described as below |  |
|  |  |  |  |
| **Major gastrointestinal bleeding** | I85, K22.1, I22.8, K25.0, K25.2, K25.4, K25.6, K26.0, K26.2, K26.4, K26.6, K27.0, K27.2, K27.4, K27.6, K28.0, K28.2, K28.4, K28.6, K29.01, K29.21, K29.31, K29.41, K29.51, K29.61, K29.71, K29.81, K29.91, K31.8, K92.0, K92.1, K92.2, K55.2, K57.0, K57.1, K57.2, K57.3, K57.4, K57.5, K57.8, K57.9, K62.5, K66.1 | Primary diagnosis, admission≥1 or red blood cell transfusion≥1 |  |
| **Intracranial hemorrhage** | I60-62 | Primary diagnosis, admission ≥1 (≥3 days), and brain imaging (CT or MRI) ≥1 |  |
| **Extracranial/unclassified major bleeding** | D62, H05.2, H35.6, H43.1, J94.2, M25.0, R04 | Primary diagnosis and [admission≥1 or red blood cell transfusion≥1] |  |
| **All-cause death** | Death from any cause | Data from Statistics Korea linked with the Korean NHIS database. |  |
| **Net clinical outcome** | Composite of ischemic stroke, major bleeding, and all-cause death |  |  |

Abbreviations: ICD-10-CM, International Classification of Diseases 10th Clinical Modification; NHIS, national health insurance service.

**Supplementary Table 2. Definition of Charlson comorbidity index**

| **Category** | **Weights** | **Disease** | **ICD-10-CM code** |
| --- | --- | --- | --- |
| **Myocardial infarction** | 1 | Acute myocardial infarction | I21 |
|  |  | Subsequent myocardial infarction | I22 |
| **Congestive heart failure** | 1 | Heart Failure | I50 |
| **Peripheral vascular disease** | 1 | Atherosclerosis | I70 |
|  |  | Other peripheral vascular disease | I73 |
| **Cerebrovascular disease** | 1 | Transient cerebral ischemic attacks and related syndromes | G45 |
|  |  | Vascular syndromes of brain in cerebrovascular diseases | G46 |
|  |  | Retinal vascular occlusion | H34 |
|  |  | Cerebrovascular disease | I60-I69 |
| **Dementia** | 1 | Dementia in Alzheimer disease | F00 |
|  |  | Vascular dementia | F01 |
|  |  | Dementia in other disease classified elsewhere | F02 |
|  |  | Unspecified dementia | F03 |
| **Chronic pulmonary disease** | 1 | Chronic lower respiratory diseases | J40-J47 |
|  |  | Lung disease due to external agents | J60-J67 |
| **Rheumatic disease** | 1 | Rheumatoid arthritis with rheumatoid factor | M05 |
| **(connective tissue disorder)** |  | Felty's syndrome | M05.0 |
|  |  | Rheumatoid lung disease with rheumatoid arthritis | M05.1 |
|  |  | Rheumatoid vasculitis with rheumatoid arthritis | M05.2 |
|  |  | Rheumatoid heart disease with rheumatoid arthritis | M05.3 |
|  |  | Rheumatoid myopathy with rheumatoid arthritis | M05.4 |
|  |  | Rheumatoid polyneuropathy with rheumatoid arthritis | M05.5 |
|  |  | Rheumatoid arthritis with involvement of other organs and systems | M05.6 |
|  |  | Rheumatoid arthritis with rheumatoid factor without organ or systems involvement | M05.7 |
|  |  | Other rheumatoid arthritis with rheumatoid factor | M05.8 |
|  |  | Rheumatoid arthritis without rheumatoid factor | M05.9 |
|  |  | Adult-onset Still's disease | M06.1 |
|  |  | Rheumatoid bursitis | M06.2 |
|  |  | Rheumatoid nodule | M06.3 |
|  |  | Inflammatory polyarthropathy | M06.4 |
|  |  | Other specified rheumatoid arthritis | M06.8 |
|  |  | Rheumatoid arthritis, unspecified | M06.9 |
|  |  | Giant cell arteritis with polymyalgia rheumatica | M31.5 |
|  |  | Systemic lupus erythematosus (SLE) | M32 |
|  |  | Drug-induced SLE | M32.0 |
|  |  | SLE with organ or system involvement | M32.1 |
|  |  | Other forms of SLE | M32.8 |
|  |  | SLE, unspecified | M32.9 |
|  |  | Dermatopolymyositis | M33 |
|  |  | Juvenile dermatomyositis | M33.0 |
|  |  | Other dermatomyositis | M33.1 |
|  |  | Polymyositis | M33.2 |
|  |  | Dermatopolymyositis, unspecified | M33.9 |
|  |  | Systemic sclerosis [scleroderma] | M34 |
|  |  | Progressive systemic sclerosis | M34.0 |
|  |  | CR(E)ST syndrome | M34.1 |
|  |  | Systemic sclerosis induced by drug and chemical | M34.2 |
|  |  | Other forms of systemic sclerosis | M34.8 |
|  |  | Systemic sclerosis, unspecified | M34.9 |
|  |  | Other overlap syndromes | M35.1 |
|  |  | Polymyalgia rheumatica | M35.3 |
|  |  | Dermato(poly)myositis in neoplastic disease | M36.0 |
| **Peptic ulcer disease** | 1 | Gastric ulcer | K25 |
|  |  | Duodenal ulcer | K26 |
|  |  | Peptic ulcer, site unspecified | K27 |
|  |  | Gastrojejunal ulcer | K28 |
| **Mild liver disease** | 1 | Chronic viral hepatitis | B18 |
|  |  | Alcoholic fatty liver | K70.0- K70.3, K70.9 |
|  |  | Alcoholic hepatitis |  |
|  |  | Alcoholic fibrosis and sclerosis of liver |  |
|  |  | Alcoholic cirrhosis of liver |  |
|  |  | Alcoholic liver disease, unspecified |  |
|  |  | Toxic liver disease with chronic persistent hepatitis | K71.3- K71.5, K71.7 |
|  |  | Toxic liver disease with chronic lobular hepatitis |  |
|  |  | Toxic liver disease with chronic active hepatitis |  |
|  |  | Toxic liver disease with fibrosis and cirrhosis of liver |  |
|  |  | Chronic hepatitis, not elsewhere classified | K73 |
|  |  | Fibrosis and cirrhosis of liver | K74 |
|  |  | Fatty (change of) liver, not elsewhere classified | K76.0-K76.4, K76.8, K76.9 |
|  |  | Nonalcoholic fatty liver disease |  |
|  |  | Central hemorrhagic necrosis of liver |  |
|  |  | Infarction of liver |  |
|  |  | Hepatic angiomatosis |  |
|  |  | Other specified disease of liver |  |
|  |  | Simple cyst of liver |  |
|  |  | Focal nodular hyperplasia of liver |  |
|  |  | Hepatoptosis |  |
|  |  | Liver disease, unspecified |  |
|  |  | Liver transplant status | Z94.4 |
| **Diabetes without chronic** | 1 | with coma | E10.0, 10.1, 10.6, 10.8, 10.9 |
| **complication** |  | with ketoacidosis | E11.0, 11.1, 11.6, 11.8, 11.9 |
|  |  | with other specified complications | E12.0, 12.1, 12.6, 12.8, 12.9 |
|  |  | with unspecified complications | E13.0, 13.1, 13.6, 13.8, 13.9 |
|  |  | without complications | E14.0, 14.1, 14.6, 14.8, 14.9 |
| **Diabetes with chronic** | 2 | with renal complications | E10.2, 10.3, 10.4, 10.5, 10.7 |
| **complication** |  | with ophthalmic complications | E11.2, 11.3, 11.4, 11.5, 11.7 |
|  |  | with neurologic complications | E12.2, 12.3, 12.4, 12.5, 12.7 |
|  |  | with peripheral circulatory complications | E13.2, 13.3, 13.4, 13.5, 13.7 |
|  |  | with multiple complications | E14.2, 14.3, 14.4, 14.5, 14.7 |
| **Hemi/paraplegia** | 2 | Tropical spastic paraplegia | G04.1 |
|  |  | Hereditary spastic paraplegia | G11.4 |
|  |  | Spastic quadriplegic cerebral palsy | G80.0 |
|  |  | Spastic diplegic cerebral palsy | G80.1 |
|  |  | Spastic hemiplegic cerebral palsy | G80.2 |
|  |  | Flaccid hemiplegia | G81.0 |
|  |  | Spastic hemiplegia | G81.1 |
|  |  | Hemiplegia, unspecified | G81.9 |
|  |  | Flaccid paraplegia | G82.0 |
|  |  | Spastic paraplegia | G82.1 |
|  |  | Paraplegia, unspecified | G82.2 |
|  |  | Flaccid tetraplegia | G82.3 |
|  |  | Spastic tetraplegia | G82.4 |
|  |  | Tetraplegia, unspecified | G82.5 |
|  |  | Diplegia of upper limbs | G83.0 |
|  |  | Paralytic syndrome, unspecified | G83.9 |
| **Renal disease** | 2 | Hypertensive renal disease | I12 |
|  |  | Hypertensive heart and renal disease with renal failure | I13.1 |
|  |  | Chronic nephritic syndrome | N03 |
|  |  | Unspecified nephritic syndrome | N05 |
|  |  | Chronic kidney disease | N18 |
|  |  | Unspecified kidney failure | N19 |
|  |  | Disorders resulting from impaired renal tubular function | N25 |
|  |  | Care involving dialysis | Z49 |
|  |  | Transplanted organ and tissue status - kidney | Z94.0 |
|  |  | Dependence on renal dialysis | Z99.2 |
| **Cancer** | 2 | Any tumor, malignant neoplasm | C00-76, C97 |
|  |  | Any tumor, in situ neoplasm | D00-09 |
|  |  | Any tumor, Benign neoplasm | D10-36 |
|  |  | Any tumor, Neoplasm of unknown behavior | D37-48 |
|  |  | Leukemia | C91-95 |
|  |  | Lymphoma | C81-86 |
| **Metastatic cancer** | 3 | Metastatic solid tumor | C77-80 |
| **Moderate to severe** | 3 | Esophageal varices | I85 |
| **liver disease** |  | Gastric varices | I86.4 |
|  |  | Esophageal varices without bleeding in diseases classified elsewhere | I98.2 |
|  |  | Alcoholic hepatic failure | K70.4 |
|  |  | Toxic liver disease with hepatic necrosis | K71.1 |
|  |  | Hepatic failure (acute/chronic) due to drugs |  |
|  |  | Chronic hepatic failure | K72.1, K72.9 |
|  |  | Hepatic failure, unspecified |  |
|  |  | Hepatic veno-occlusive disease | K76.5-K76.7 |
|  |  | Portal hypertension |  |
|  |  | Hepatorenal syndrome |  |
| **Human immunodeficiency** | 6 | HIV disease resulting in infectious and parasitic diseases | B20 |
| **Virus (HIV)** |  | HIV disease resulting in malignant neoplasm | B21 |
|  |  | HIV disease resulting in other specified diseases | B22 |
|  |  | HIV disease resulting in other conditions | B23 |

**Supplementary Table 3.** **Calculation of the hospital Frailty risk score. The score is an aggregate of 109 ICD-10 diagnostic codes that were found to be associated with frailty risk**

| ICD Code | Points awarded | ICD Description |  |
| --- | --- | --- | --- |
|  |  |  |  |
|  |  |  |  |
| K59 | 1.8 | Other functional intestinal disorders |  |
| G81 | 4.4 | Hemiplegia |  |
| N39 | 3.2 | Other disorders of urinary system (includes urinary tract infection and urinary incontinence) |  |
| F00 | 7.1 | Dementia in Alzheimer's disease |  |
| I69 | 3.7 | Sequelae of cerebrovascular disease (secondary codes) |  |
| E87 | 2.3 | Other disorders of fluid, electrolyte and acid base balance |  |
| I63 | 0.8 | Cerebral Infarction |  |
| M81 | 1.4 | Osteoporosis without pathological fracture |  |
| J18 | 1.1 | Pneumonia, organism unspecified |  |
| I67 | 2.6 | Other cerebrovascular diseases |  |
| S06 | 2.4 | Intracranial injury |  |
| M25 | 2.3 | Other joint disorders, not elsewhere classified |  |
| R31 | 3 | Unspecified hematuria |  |
| A09 | 1.1 | Diarrhea and gastroenteritis of presumed infectious origin |  |
| S22 | 1.8 | Fracture of rib(s), sternum and thoracic spine |  |
| G40 | 1.5 | Epilepsy |  |
| M79 | 1.1 | Other soft tissue disorders, not elsewhere classified |  |
| R94 | 1.4 | Abnormal results of function studies |  |
| M19 | 1.5 | Other arthrosis |  |
| S00 | 3.2 | Superficial injury of head |  |
| F03 | 2.1 | Unspecified dementia |  |
| L03 | 2 | Cellulitis |  |
| S32 | 1.4 | Fracture of lumbar spine and pelvis |  |
| R55 | 1.8 | Syncope and collapse |  |
| N17 | 1.8 | Acute renal failure |  |
| G45 | 1.2 | Transient cerebral ischemic attacks and related syndromes |  |
| F05 | 3.2 | Delirium, not induced by alcohol and other psychoactive substances |  |
| S01 | 1.1 | Open wound of head |  |
| L89 | 1.7 | Decubitus ulcer |  |
| F01 | 2 | Vascular dementia |  |
| R11 | 0.3 | Nausea and vomiting |  |
| E86 | 2.3 | Volume depletion |  |
| E05 | 0.9 | Thyrotoxicosis [hyperthyroidism] |  |
| M48 | 0.5 | Spinal stenosis (secondary code only) |  |
| R56 | 2.6 | Convulsions, not elsewhere classified |  |
| F32 | 0.5 | Depressive episode |  |
| N18 | 1.4 | Chronic renal failure |  |
| N28 | 1.3 | Other disorders of kidney and ureter, not elsewhere classified |  |
| S80 | 2 | Superficial injury of lower leg |  |
| R26 | 2.6 | Abnormalities of gait and mobility |  |
| R00 | 0.7 | Abnormalities of heart beat |  |
| A41 | 1.6 | Other septicemia |  |
| K26 | 1.6 | Duodenal ulcer |  |
| K92 | 0.8 | Other diseases of digestive system |  |
| R47 | 1 | Speech disturbances, not elsewhere classified |  |
| S72 | 1.4 | Fracture of femur |  |
| D64 | 0.4 | Other anemia |  |
| S42 | 2.3 | Fracture of shoulder and upper arm |  |
| R40 | 2.5 | Somnolence, stupor and coma |  |
| R41 | 2.7 | Other symptoms and signs involving cognitive functions and awareness |  |
| I95 | 1.6 | Hypotension |  |
| R13 | 0.8 | Dysphagia |  |
| G30 | 4 | Alzheimer's disease |  |
| E16 | 1.4 | Other disorders of pancreatic internal secretion |  |
| A04 | 1.1 | Other bacterial intestinal infections |  |
| M80 | 0.8 | Osteoporosis with pathological fracture |  |
| G20 | 1.8 | Parkinson's disease |  |
| N20 | 0.7 | Calculus of kidney and ureter |  |
| G31 | 1.2 | Other degenerative diseases of nervous system, not elsewhere classified |  |
| R63 | 0.9 | Symptoms and signs concerning food and fluid intake |  |
| J69 | 1 | Pneumonitis due to solids and liquids |  |
| B96 | 2.9 | Other bacterial agents as the cause of diseases classified to other chapters (secondary code) |  |
| J96 | 1.5 | Respiratory failure, not elsewhere classified |  |
| N19 | 1.6 | Unspecified renal failure |  |
| K52 | 0.3 | Other noninfective gastroenteritis and colitis |  |
| E53 | 1.9 | Deficiency of other B group vitamins |  |
| R50 | 0.1 | Fever of unknown origin |  |
| F10 | 0.7 | Mental and behavioral disorders due to use of alcohol |  |
| H91 | 0.9 | Other hearing loss |  |
| E83 | 0.4 | Disorders of mineral metabolism |  |
| M15 | 0.4 | Polyarthrosis |  |
| R29 | 3.6 | Other symptoms and signs involving the nervous and musculoskeletal systems (R29.6 Tendency to fall) |  |
| R33 | 1.3 | Retention of urine |  |
| Z93 | 1 | Artificial opening status |  |
| E55 | 1 | Vitamin D deficiency |  |
| Z22 | 1.7 | Carrier of infectious disease |  |
| R32 | 1.2 | Unspecified urinary incontinence |  |
| L08 | 0.4 | Other local infections of skin and subcutaneous tissue |  |
| R79 | 0.6 | Other abnormal findings of blood chemistry |  |
| R54 | 2.2 | Senility |  |
| Z50 | 2.1 | Care involving use of rehabilitation procedures |  |
| S51 | 0.5 | Open wound of forearm |  |
| B95 | 1.7 | Streptococcus and staphylococcus as the cause of diseases classified to other chapters |  |
| R45 | 1.2 | Symptoms and signs involving emotional state |  |
| M41 | 0.9 | Scoliosis |  |
| S09 | 1.2 | Other and unspecified injuries of head |  |
| H54 | 1.9 | Blindness and low vision |  |
| R02 | 1 | Gangrene, not elsewhere classified |  |
| L97 | 1.6 | Ulcer of lower limb, not elsewhere classified |  |
| W01 | 0.9 | Fall on same level from slipping, tripping and stumbling |  |
| J22 | 0.7 | Unspecified acute lower respiratory infection |  |
| W18 | 2.1 | Other fall on same level |  |
| R44 | 1.6 | Other symptoms and signs involving general sensations and perceptions |  |
| W19 | 3.2 | Unspecified fall |  |
| R69 | 1.3 | Unknown and unspecified causes of morbidity |  |
| T83 | 2.4 | Complications of genitourinary prosthetic devices, implants and grafts |  |
| Z87 | 1.5 | Personal history of other diseases and conditions |  |
| Z75 | 2 | Problems related to medical facilities and other health care |  |
| X59 | 1.5 | Exposure to unspecified factor |  |
| W10 | 0.9 | Fall on and from stairs and steps |  |
| U80 | 0.8 | Agent resistant to penicillin and related antibiotics |  |
| Z99 | 0.8 | Dependence on enabling machines and devices |  |
| Y95 | 1.2 | Nosocomial condition |  |
| W06 | 1.1 | Fall involving bed |  |
| Y84 | 0.7 | Other medical procedures as the cause of abnormal reaction of the patient |  |
| Z91 | 0.5 | Personal history of risk-factors, not elsewhere classified |  |
| Z74 | 1.1 | Problems related to care-provider dependency |  |
| Z73 | 0.6 | Problems related to life-management difficulty |  |
| Z60 | 1.8 | Problems related to social environment |  |

**Supplementary Table 4. Baseline characteristics of total study population**

|  | **Total study population** |
| --- | --- |
| **Number** | 21,468 |
| **Age, years (mean±SD)** | 85.3±4.4 |
| **Age, years (median, IQR)** | 84 (82-88) |
| **Age** |  |
| **80 <age≤ 85** | 10,908 (50.8) |
| **85 <age≤ 90** | 6898 (32.1) |
| **≥90** | 3662 (17.1) |
| **Female** | 13,743 (64.0) |
| **Charlson comorbidity score (mean±SD)** | 3.9±2.4 |
| **>3** | 11,045 (51.5) |
| **Frailty index (mean±SD)** | 9.3±7.2 |
| **CHA_2_DS_2_-VASc score (mean±SD)** | 4.4±1.1 |
| **CHA_2_DS_2_-VASc score (median, IQR)** | 4 (4-5) |
| **CHA_2_DS_2_-VASc score** |  |
| **2** | 930 (4.3) |
| **3** | 3541 (16.5) |
| **4** | 6723 (31.3) |
| **5** | 6668 (31.1) |
| **6** | 3039 (14.2) |
| **≥7** | 567 (2.6) |
| **Comorbidities** |  |
| **Hypertension** | 17,447 (81.3) |
| **Diabetes mellitus** | 5024 (23.4) |
| **Heart failure** | 8778 (40.9) |
| **Dyslipidemia** | 8229 (38.3) |
| **Prior myocardial infarction** | 1703 (7.9) |
| **Chronic kidney disease** | 3875 (18.1) |
| **Peripheral artery disease** | 5754 (26.8) |
| **Chronic obstructive pulmonary disease** | 2789 (13.0) |
| **Cancer** | 1724 (8.0) |
| **Low income** | 3391 (15.8) |
|  |  |
| **Patients with health examination data*** | 9533 (44.4) |
| **Serum creatinine (mean±SD, g/dL)** | 0.99±0.89 |
| **eGFR (mean±SD, ml/min/1.73m^2^)** | 72.9±37.3 |
| **Body weight (mean±SD, kg)** | 55.6±11.2 |
| **≤45 kg** | 2064 (21.7) |
| **45<body weight≤60 kg** | 4359 (45.7) |
| **>60 kg** | 3110 (32.6) |
| **Body mass index (mean±SD, kg/m^2^)** | 23.2±3.6 |
| **Smoking** |  |
| **Non-smoker** | 7326 (76.9) |
| **Ex-smoker** | 1610 (16.9) |
| **Current smoker** | 597 (6.3) |
| **Alcohol** |  |
| **Non** | 8101 (85.0) |
| **Mild to moderate** | 1242 (13.0) |
| **Heavy drinker** | 190 (2.0) |
| **Performing regular exercise** | 1082 (11.4) |

*The following variables were calculated among patients with available health examination data.

Continuous variables are shown as mean and standard deviation. Categorical variables are presented as percentages.

Abbreviation: IPTW, inverse probability of treatment weighting; DOAC, direct oral anticoagulant; OAC, oral anticoagulant; ASD, absolute standardized difference; IQR, interquartile range; LDL, low-density lipoprotein; SD, standard deviation; eGFR, estimated glomerular filtration rate.

**Supplementary Table 5. Baseline characteristics of non-OAC group and Warfarin group before and after IPTW**

|  |  | **Pre-IPTW** | | |  |  |  | **Post-IPTW** | | |  |
| --- | --- | --- | --- | --- | --- | --- | --- | --- | --- | --- | --- |
|  | **Non-OAC** | | **Warfarin** | **ASD** | |  | **Non-OAC** | | **Warfarin** | **ASD** | |
| **Number** | 16,575 | | 2,390 |  | |  | 16,575 | | 2,389 |  | |
| **Age, years (mean±SD)** | 85.4±4.4 | | 84.4±3.8 | 0.234 | |  | 85.3±4.4 | | 85.2±4.1 | 0.015 | |
| **Age, years (median, IQR)** | 84 (82-88) | | 84 (81-87) |  | |  | 84 (82-88) | | 84 (82-88) |  | |
| **Age** |  | |  |  | |  |  | |  |  | |
| **80 <age≤ 85** | 50.0 | | 58.4 |  | |  | 51.1 | | 50.1 |  | |
| **85 <age≤ 90** | 32.1 | | 30.5 |  | |  | 31.7 | | 33.8 |  | |
| **≥90** | 17.9 | | 11.1 |  | |  | 17.1 | | 16.1 |  | |
| **Female** | 63.4 | | 61.6 | 0.038 | |  | 63.2 | | 62.9 | 0.006 | |
| **Charlson comorbidity score (mean±SD)** | 3.9±2.4 | | 4.0±2.4 | 0.033 | |  | 3.9±2.4 | | 3.9±2.4 | 0.025 | |
| **Frailty index (mean±SD)** | 9.4±72 | | 8.2±6.7 | 0.163 | |  | 9.2±7.2 | | 9.3±7.3 | 0.010 | |
| **CHA_2_DS_2_-VASc score (mean±SD)** | 4.4±1.1 | | 4.6±1.1 | 0.188 | |  | 4.4±1.1 | | 4.4±1.1 | 0.002 | |
| **CHA_2_DS_2_-VASc score (median, IQR)** | 4 (4-5) | | 5 (4-5) |  | |  | 4 (4-5) | | 4 (4-5) |  | |
| **CHA_2_DS_2_-VASc score** |  | |  |  | |  |  | |  |  | |
| **2** | 4.9 | | 2.7 |  | |  | 4.6 | | 4.1 |  | |
| **3** | 17.3 | | 13.7 |  | |  | 16.9 | | 17.5 |  | |
| **4** | 31.7 | | 30.2 |  | |  | 31.5 | | 32.1 |  | |
| **5** | 30.3 | | 32.6 |  | |  | 30.7 | | 29.3 |  | |
| **6** | 13.3 | | 17.6 |  | |  | 13.7 | | 14.6 |  | |
| **≥7** | 2.5 | | 3.2 |  | |  | 2.6 | | 2.5 |  | |
| **Comorbidities** |  | |  |  | |  |  | |  |  | |
| **Hypertension** | 80.3 | | 87.0 | 0.179 | |  | 81.1 | | 81.6 | 0.011 | |
| **Diabetes mellitus** | 23.3 | | 27.7 | 0.101 | |  | 23.8 | | 24.5 | 0.015 | |
| **Heart failure** | 37.7 | | 49.3 | 0.233 | |  | 39.2 | | 38.7 | 0.010 | |
| **Dyslipidemia** | 36.9 | | 42.4 | 0.112 | |  | 37.6 | | 38.0 | 0.007 | |
| **Prior myocardial infarction** | 8.1 | | 9.3 | 0.043 | |  | 8.3 | | 8.2 | 0.001 | |
| **Chronic kidney disease** | 18.6 | | 20.2 | 0.079 | |  | 18.8 | | 19.0 | 0.042 | |
| **Peripheral artery disease** | 26.6 | | 26.2 | 0.010 | |  | 26.6 | | 26.6 | 0.001 | |
| **Chronic obstructive pulmonary disease** | 12.8 | | 15.3 | 0.072 | |  | 13.1 | | 12.8 | 0.009 | |
| **Cancer** | 8.1 | | 6.8 | 0.049 | |  | 7.9 | | 8.5 | 0.020 | |
| **Low income** | 16.0 | | 15.9 | 0.002 | |  | 16.0 | | 15.7 | 0.009 | |
|  |  | |  |  | |  |  | |  |  | |
| **Patients with health examination data*** | 45.4 | | 41.2 |  | |  |  | |  |  | |
| **eGFR (mean±SD, ml/min/1.73m^2^)** | 72.7±39.9 | | 69.1±21.3 | 0.110 | |  | 72.6±40.2 | | 69.3±20.9 | 0.103 | |
| **Body weight (mean±SD, kg)** | 55.4±11.1 | | 56.9±10.8 | 0.134 | |  | 55.5±11.1 | | 56.1±10.6 | 0.061 | |
| **≤45 kg** | 21.6 | | 15.8 |  | |  | 21.2 | | 18.1 |  | |
| **45<body weight≤60 kg** | 46.5 | | 48.2 |  | |  | 46.5 | | 47.7 |  | |
| **>60 kg** | 31.9 | | 36.0 |  | |  | 32.3 | | 34.2 |  | |
| **Smoking** |  | |  | 0.060 | |  |  | |  | 0.022 | |
| **Non-smoker** | 76.6 | | 74.1 |  | |  | 76.5 | | 75.6 |  | |
| **Ex-smoker** | 17.0 | | 19.2 |  | |  | 17.1 | | 18.3 |  | |
| **Current smoker** | 6.4 | | 6.7 |  | |  | 6.4 | | 6.1 |  | |
| **Alcohol** |  | |  | 0.083 | |  |  | |  | 0.068 | |
| **Non** | 84.9 | | 81.8 |  | |  | 84.8 | | 82.3 |  | |
| **Mild to moderate** | 13.1 | | 14.8 |  | |  | 13.2 | | 14.3 |  | |
| **Heavy drinker** | 2.0 | | 3.4 |  | |  | 2.0 | | 3.4 |  | |
| **Performing regular exercise** | 11.4 | | 12.4 | 0.002 | |  | 11.4 | | 12.1 | 0.022 | |

*The following variables were calculated among patients with available health examination data.

Continuous variables are shown as mean and standard deviation. Categorical variables are presented as percentages.

Abbreviation: IPTW, inverse probability of treatment weighting; DOAC, direct oral anticoagulant; OAC, oral anticoagulant; ASD, absolute standardized difference; IQR, interquartile range; LDL, low-density lipoprotein; SD, standard deviation; eGFR, estimated glomerular filtration rate

**Supplementary Figure1. Subgroup analyses according to the age, sex, CHA₂DS₂-VASc score, GFR, previous history of GI bleeding, body weight, NSAID use, and antiplatelet agent use.**


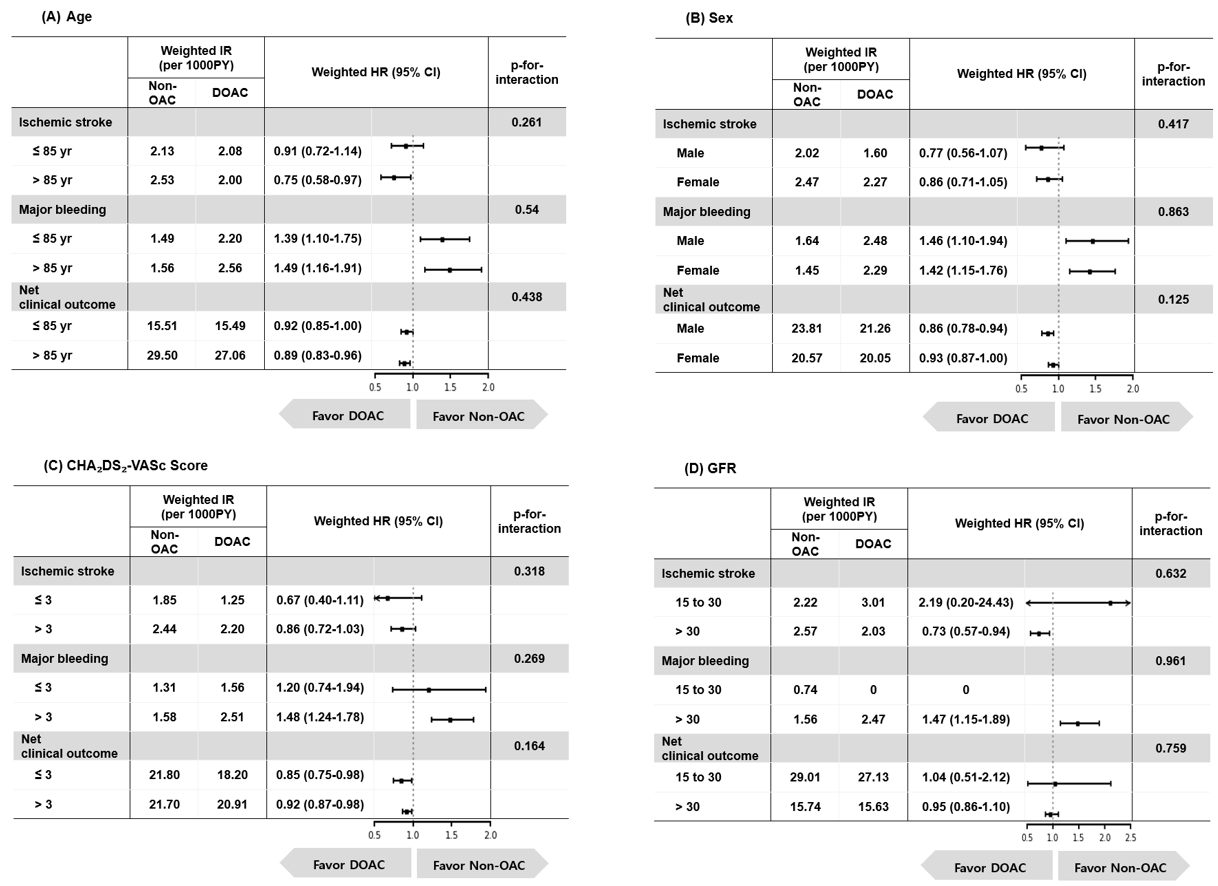


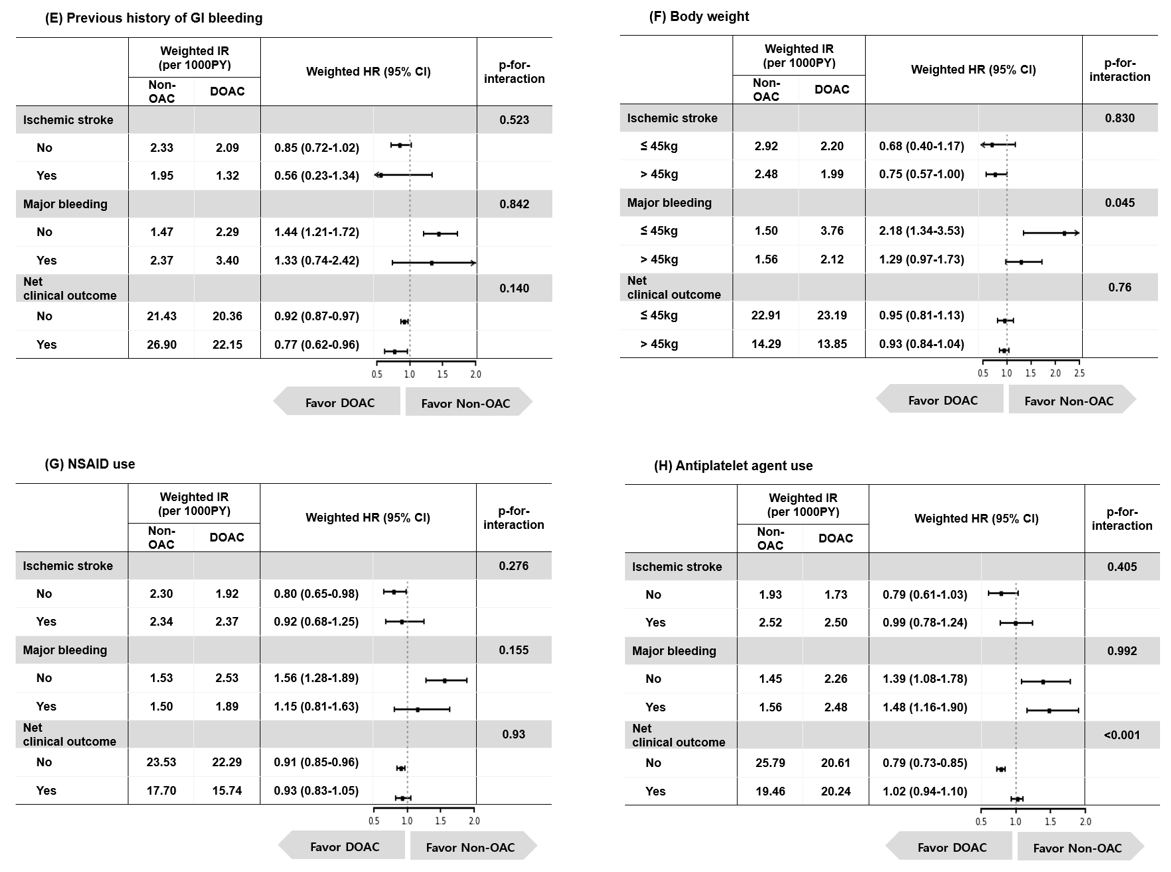


Abbreviation: IR, incidence rate; PY, person-years; HR, hazard ratio; CI, confidence interval; OAC, oral anticoagulant; DOAC, direct oral anticoagulant; GFR, glomerular filtration rate; GI, gastrointestinal; NSAID, nonsteroidal anti-inflammatory drug.

**Supplementary Figure 2. Hazard ratios of ischemic stroke, major bleeding, all-cause death, and net clinical outcome in 4 direct oral anticoagulants versus Non-OAC group**


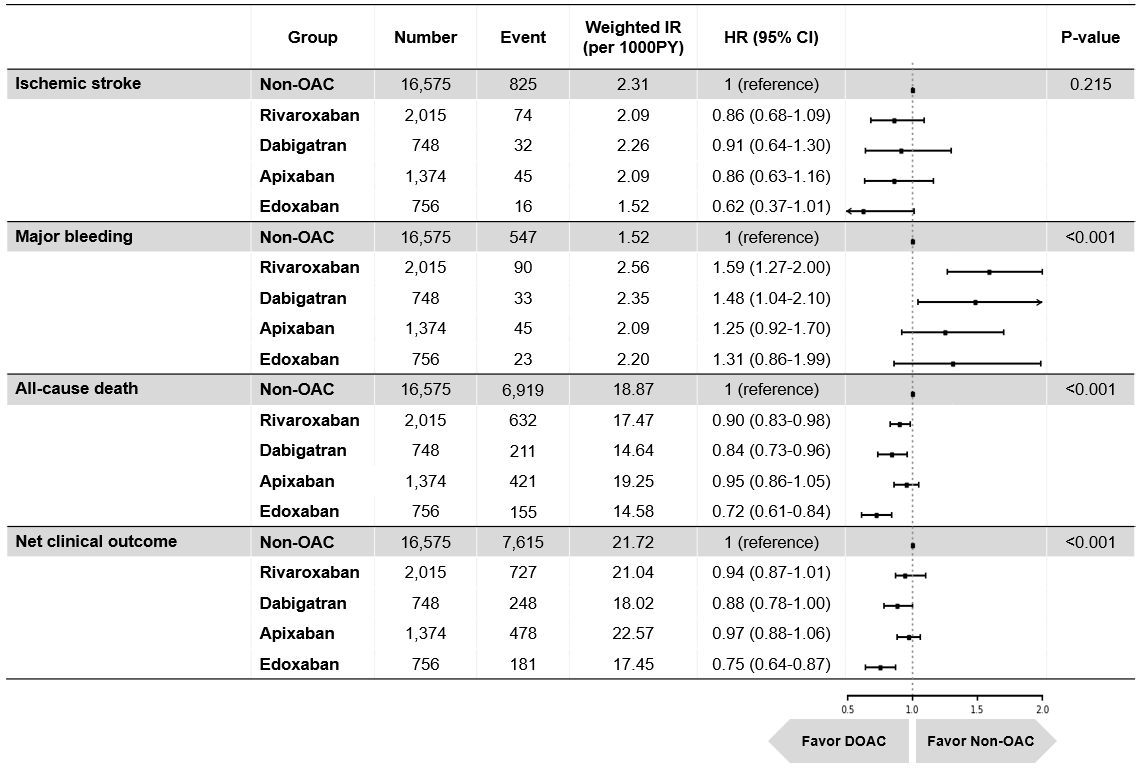


Abbreviation: IR, incidence rate; PY, person-years; HR, hazard ratio; CI, confidence interval; OAC, oral anticoagulant; DOAC, direct oral anticoagulant

**Supplementary Figure 3. Hazard ratios of ischemic stroke, major bleeding, all-cause death, and net clinical outcome by DOAC dose**


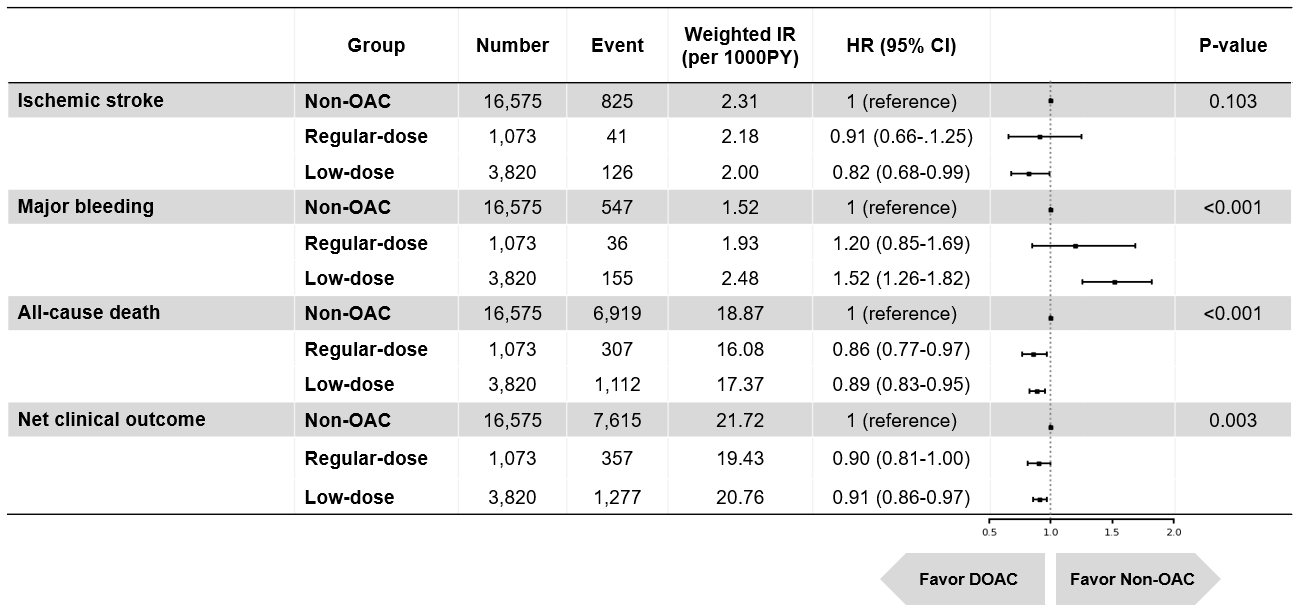


Abbreviation: IR, incidence rate; PY, person-years; HR, hazard ratio; CI, confidence interval; OAC, oral anticoagulant; DOAC, direct oral anticoagulant

**Supplementary Figure 4. Anticoagulant prescription trends over one year in the ELDERCARE-AF-like cohort**


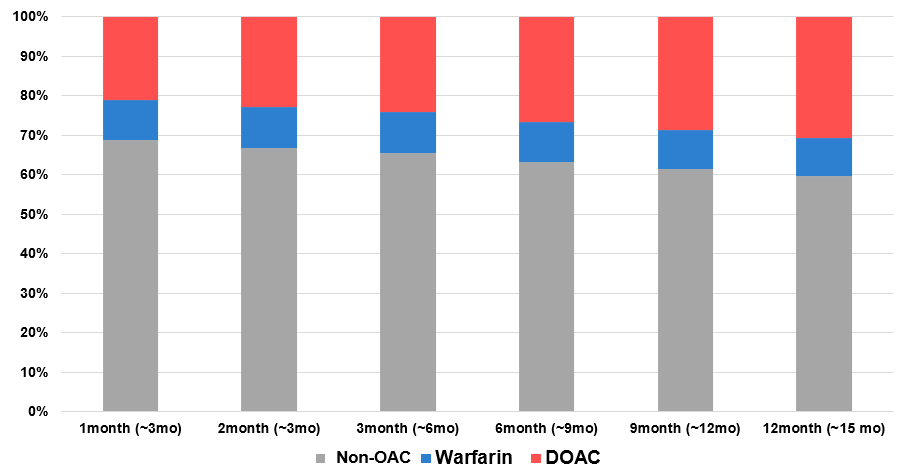


Abbreviation: OAC, oral anticoagulant; DOAC, direct oral anticoagulant
